# Synergistic Coupling of Thermal Decomposition–Ammonia Dissolution and Segmented Crystallization for High-Purity Ammonium Paratungstate

**DOI:** 10.3390/ma19173788

**Published:** 2026-09-06

**Authors:** Lyuming Chen, Lairong Xiao, Zhengda He, Yuxiang Jiang, Sainan Liu, Shaohao Li, Qingkui Li, Yongli Li, Xiaojun Zhao, Zhenyang Cai

**Affiliations:** 1 School of Materials Science and Engineering, Central South University, Changsha 410083, China; 253102054@csu.edu.cn (L.C.); xiaolr@csu.edu.cn (L.X.); hezhenda2004@163.com (Z.H.); jiangyuxiang1999@163.com (Y.J.); lishaohao2022@163.com (S.L.); 2 School of Minerals Processing and Bioengineering, Central South University, Changsha 410083, China; lsn@csu.edu.cn; 3 School of Materials Science and Engineering, Zhengzhou University, Zhengzhou 450001, China; qkli491@zzu.edu.cn (Q.L.); liyongli@zzu.edu.cn (Y.L.); 4 State Key Laboratory of Powder Metallurgy, Central South University, Changsha 410083, China

**Keywords:** segmented evaporative crystallization, ammonium paratungstate (APT), thermal decomposition–ammonia dissolution, impurity purification

## Abstract

To address the escalating demand for ultra-high-purity tungsten in advanced applications such as semiconductor targets and nuclear-grade shielding, this study synergistically coupled the processes of thermal decomposition, ammonia dissolution, and segmented evaporation crystallization. The optimal parameters for the thermal decomposition and ammonia dissolution stages were subsequently identified through systematic optimization. By independently regulating the nucleation and crystal growth processes during the crystallization of ammonium paratungstate (APT), the limitation of traditional methods, which require multiple crystallization cycles to achieve high purity, is effectively overcome. Experimental results demonstrated that under optimized conditions—thermal decomposition at ~280 °C and ammonia dissolution at 90 °C—high-purity APT (4N5 grade, total impurities < 50 ppm) was achieved in a single crystallization cycle. Furthermore, under segmented crystallization conditions (nucleation at 80 °C with a stirring speed of 1.26 m/s and growth at 90 °C with a stirring speed of 1.09 m/s), the product exhibited an average particle size of 34.43 μm and a direct recovery efficiency of 73.1%. By suppressing burst nucleation and reducing impurity adsorption, this process provides a critical technological pathway for large-scale production of ultra-high-purity tungsten materials.

## 1. Introduction

As a vital nonrenewable strategic metal, tungsten boasts the highest melting point (3422 °C) and ranks among metals with the highest boiling points (5927 °C) across all elements. Its exceptional property profile—including high hardness, substantial density, superior high-temperature strength, and robust chemical stability—underpins the extensive utilization of tungsten and its alloys across aerospace, automotive, chemical, and other key industrial sectors [1,2,3,4,5].

APT functions as a critical intermediate in tungsten extraction, serving as a pivotal link between upstream and downstream tungsten metal production. Given that the chemical purity of APT directly governs the quality of final tungsten products [6], the synthesis of high-purity APT constitutes an indispensable prerequisite for manufacturing ultra-high-purity tungsten materials. High-purity tungsten produced by chemical vapor deposition (CVD) has demonstrated excellent mechanical properties, with a dynamic yield strength of more than 2000 MPa [7], highlighting the performance benefits of using ultrapure tungsten feedstock.

In recent years, tightening market specifications have imposed increasingly stringent limits on impurity elements such as Na, K, Mo, and S in APT. To address this challenge, researchers have explored targeted purification strategies: Zhao et al. [8] developed a novel sodium-selective adsorbent to tackle high sodium content in APT crystals. This adsorbent effectively removes trace sodium from ammonium tungstate solutions, achieving over 97% precipitation rate in industrial applications. Similarly, Wan et al. [9,10,11] employed activated carbon adsorption to reduce S and Mo impurities in ammonium tungstate solutions. Sun et al. [12] investigated selective precipitation for Mo removal from tungstate solutions, while Xie et al. [13] combined ion exchange with ore blending and alkaline filtration to eliminate trace Na/K impurities. In addition to these chemical purification strategies, studies on tungsten purification processes have also revealed that different impurities may follow distinct removal mechanisms depending on their physicochemical characteristics [14]. Despite their efficacy in removing individual impurities, these methods suffer from two critical limitations: inconsistent performance in removing non-targeted contaminants and inherent complexity in process integration [13,14,15,16].

Wan et al. [17] evaluated evaporative crystallization—a straightforward technique—for preparing high-purity APT. Their results indicated that this method partially eliminates K, Na, S, and Mo impurities but fails to meet the stringent requirements for ultra-high-purity APT. To address this purity gap, industrial production often employs multiple cycles of thermal decomposition–ammonia dissolution–evaporative crystallization to improve purity, though this approach significantly elevates production costs [18].

Despite these advancements, the reliance on multiple purification cycles continues to impose a significant cost burden on ultra-high-purity APT production [19]. Building on insights into the crystallization kinetics of tungstate solutions, an integrated process combining optimized thermal decomposition–ammonia dissolution with segmented evaporative crystallization is developed. Thermal decomposition at ~280 °C generates highly soluble intermediates, which are subsequently dissolved in ammonia at 90 °C to yield a homogeneous feed [20]. Controlled nucleation at 80 °C and 1.26 m/s stirring speed minimizes fine-crystal formation and unintended impurity uptake, whereas targeted crystal growth at 90 °C and 1.09 m/s stirring speed promotes the development of coarse, smooth, low-defect crystals. Through this stage-specific modulation of supersaturation, temperature, and hydrodynamics, one-step synthesis of ultra-high-purity APT is achieved.

## 2. Materials and Experiments

### 2.1. Principle

Evaporative crystallization for APT preparation relies on continuously removing free ammonia and water from an ammonium tungstate solution during heating. As ammonia concentration gradually decreases, the poorly soluble APT precipitates via the following reaction [21]:

(1) $$12{\left({NH}_{4}\right)}_{2}WO_{4}\left(aq\right)=5{\left({NH}_{4}\right)}_{2}O\cdot 12{WO}_{3}\cdot 5H_{2}O\left(s\right)+14{NH}_{3}\left(g\right)↑+2H_{2}O\left(l\right)$$

Once precipitation is complete, filtration separates APT from the mother liquor, leaving soluble impurities enriched in the liquid phase. This step effectively enhances the purity of the final APT crystals.

### 2.2. Experimental Procedure

Commercial APT powder (99.95% purity) was first subjected to thermal decomposition. The powder was placed in a muffle furnace (KSL-1100X, Hefei Kejing Materials Technology Co., Ltd., Hefei, China) and heated to 280 °C for 50 min. A targeted amount of the decomposed powder was then added to analytical-grade ammonia solution to achieve the desired initial ammonium tungstate concentration. The mixture was then transferred to a high-pressure reactor (TCYF-B-1L, Shanghai Yan Zheng Experimental Instrument Co., Ltd., Shanghai, China) equipped with a YN60BF pressure gauge, and the pressure was maintained at 1.2–1.6 MPa during the dissolution process. The mixture was stirred with a rotating agitator to facilitate dissolution. Finally, the solution was heated to 90 °C and held for 6 h to obtain a homogeneous ammonium tungstate solution [22].

**Figure 1:**
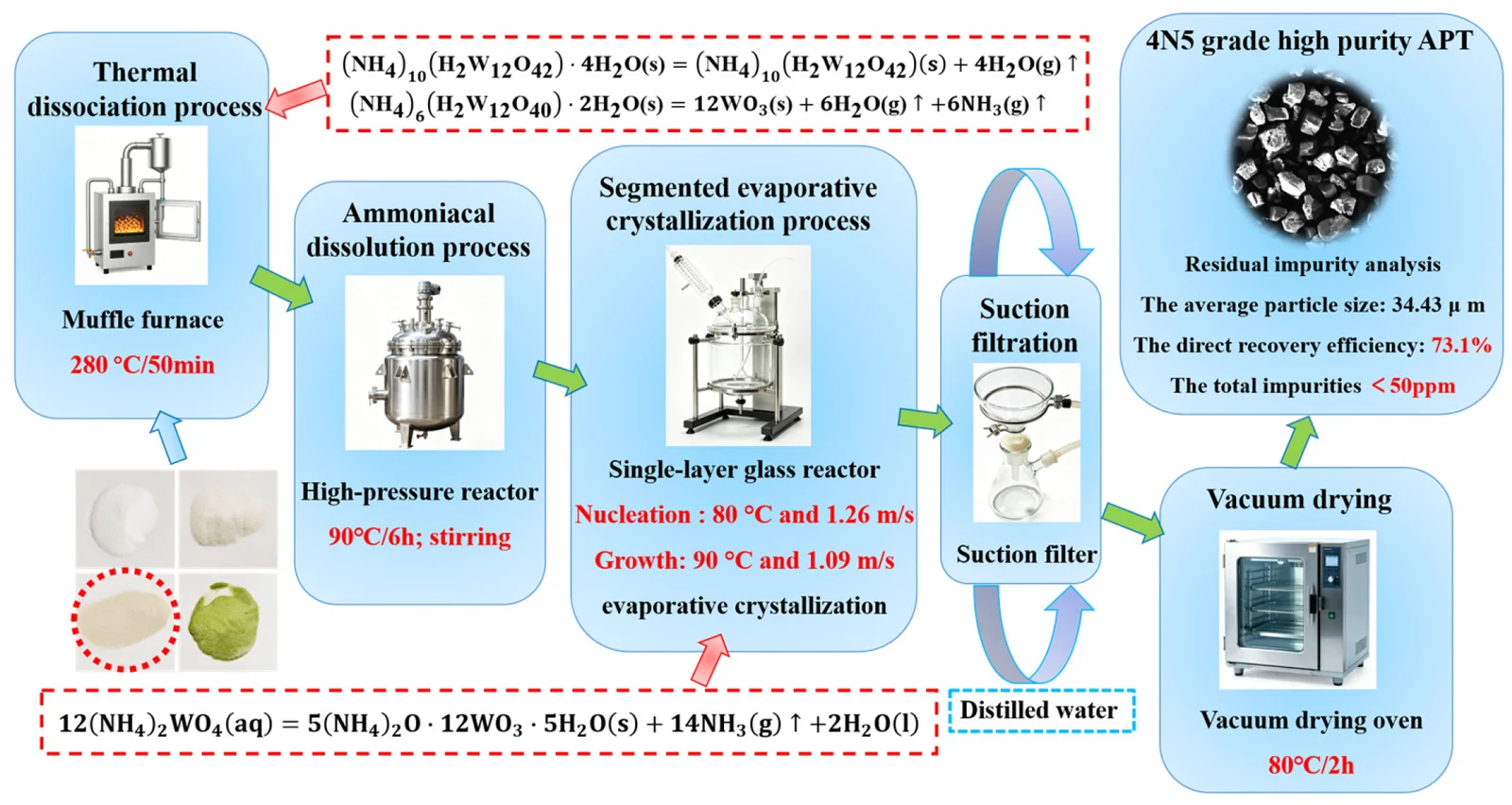
Schematic diagram of the experimental process.

The resulting solution was transferred to a single-layer glass reactor (KY-DF-5L, Shanghai Qiuzuo Scientific Instrument Co., Ltd., Shanghai, China) for evaporative crystallization. Key process parameters—including evaporation temperature, linear stirring velocity, and initial solution concentration—were tightly controlled throughout the process. Heating continued until the solution volume was reduced to approximately 1/3 of its original, maximizing direct crystallization recovery efficiency. Upon completion, the slurry of APT crystals and mother liquor was filtered by suction. The crystals were washed twice with distilled water, with suction filtering between washes. Finally, the purified APT crystals were placed in a vacuum oven and dried to produce the final product. The mother liquor was diluted to a fixed volume for the residual impurity analysis [23]. The overall experimental flow described above is illustrated in Figure 1.

### 2.3. Analysis and Characterization

The concentrations of impurity elements in the ammonium tungstate solution and the resulting mother liquor were quantified via inductively coupled plasma mass spectrometry (ICP-MS, PerkinElmer/NEXION 2000, PerkinElmer Inc., Shelton, CT, USA), providing reliable detection of trace impurities at ppm levels [24]. Recent studies have demonstrated the potential of ETV-ICP-OES (SPECTRO Analytical Instruments GmbH, Kleve, Germany) for process-accompanying analysis in high-purity tungsten production [25], while ICP-MS remains a reliable approach for solution-based impurity quantification.

The impurity precipitation rate and the mass fraction of each impurity in the APT product were calculated using the differential method [26], as defined by the following equations:

(2) $$Impurity\;precipitation\;rate:\frac{c_{0}V_{0}-c_{1}V_{1}}{c_{0}V_{0}}\times 100\%$$

(3) $$Impurity\;content\;in\;APT:\frac{c_{0}V_{0}-c_{1}V_{1}}{m_{APT}}\times 100\%$$

where c_0_ and c_1_ are the mass concentrations (g/L) of a target impurity in the initial ammonium tungstate solution and mother liquor, respectively. V_0_ and V_1_ are the corresponding volumes (L) of the initial solution and mother liquor, respectively. Here, m_APT_ is the total mass (g) of APT isolated via evaporative crystallization.

## 3. Results and Discussion

### 3.1. Composition and Microstructural Analysis of APT During Thermal Decomposition

APT exhibits low solubility in aqueous ammonia. Thermal decomposition is designed to convert APT into more ammonia-soluble compounds, a critical step for subsequent purification. To clarify the decomposition mechanism, the key reactions during thermal treatment are expressed in Equations (4) and (5):

(4) $${\left(NH_{4}\right)}_{10}\left(H_{2}W_{12}O_{42}\right)\cdot 4H_{2}O\left(s\right)={\left(NH_{4}\right)}_{10}\left(H_{2}W_{12}O_{42}\right)(s)+4H_{2}O\left(g\right)↑$$

(5) $${\left(NH_{4}\right)}_{6}\left(H_{2}W_{12}O_{40}\right)\cdot 2H_{2}O\left(s\right)=12WO_{3}\left(s\right)+6H_{2}O\left(g\right)↑+6NH_{3}\left(g\right)↑$$

**Figure 2:**
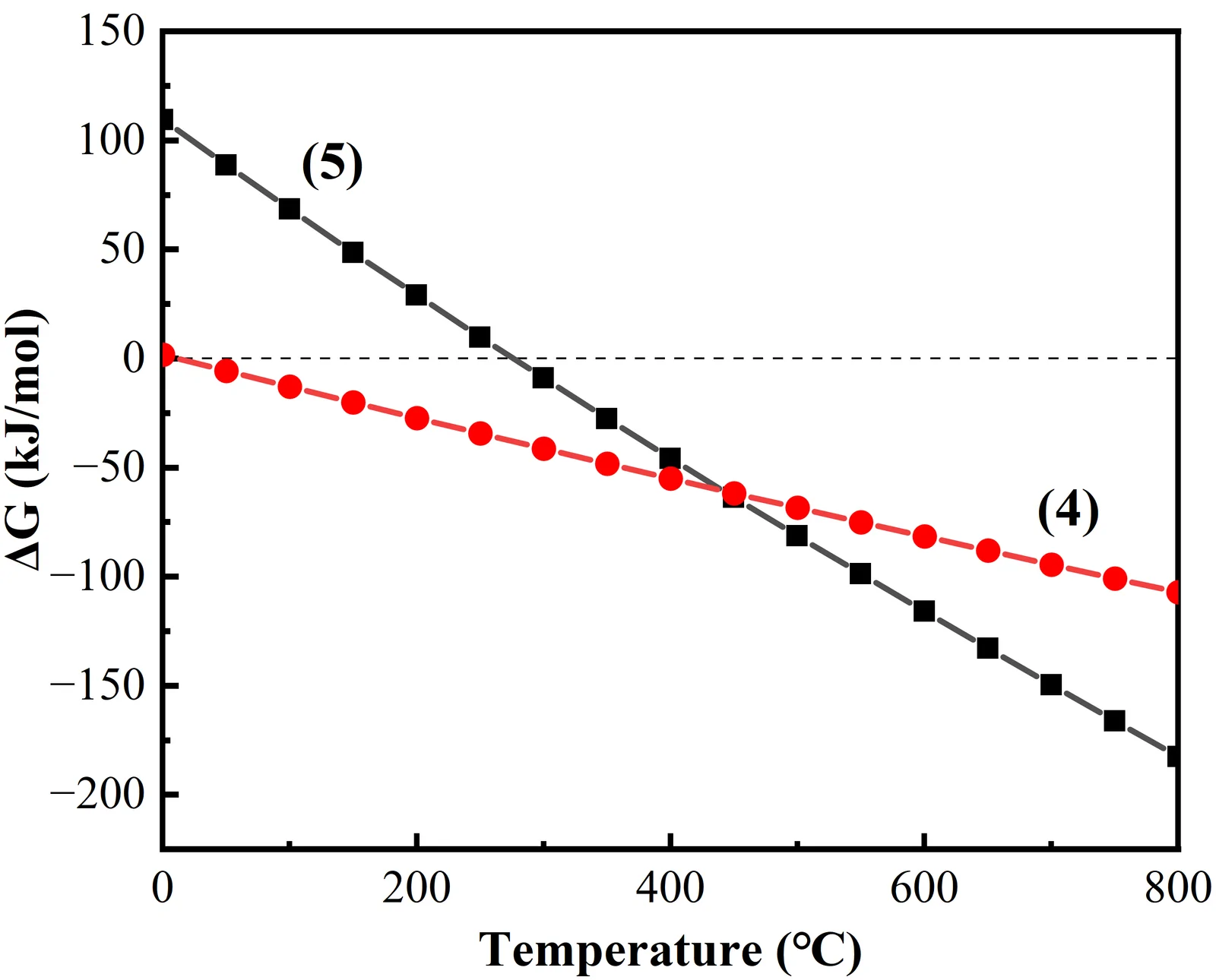
Relationship between Gibbs free energy and temperature during APT thermal decomposition.

The standard Gibbs free energy changes (∆G) for these reactions were computed using the built-in thermodynamic database of HSC Chemistry 9, and their temperature dependence is illustrated in Figure 2.

As shown in Figure 2, the standard Gibbs free energy change for Reaction (4) turns negative above 10 °C, indicating that APT initiates dehydration at temperatures exceeding this threshold. As temperature rises, ∆G becomes more negative, suggesting a trend that implies the dehydration process grows increasingly thermodynamically favorable.

For Reaction (5), ∆G becomes negative only when the temperature approaches approximately 300 °C, indicating that the conversion of APT to tungsten trioxide (WO_3_) becomes kinetically and thermodynamically viable near this temperature. Given WO_3_’s poor solubility in aqueous ammonia, it is critical to conduct the ammonia dissolution step before APT fully transforms into WO_3_. Otherwise, undissolved WO_3_ would remain as an inert residue, compromising downstream purification [27].

**Figure 3:**
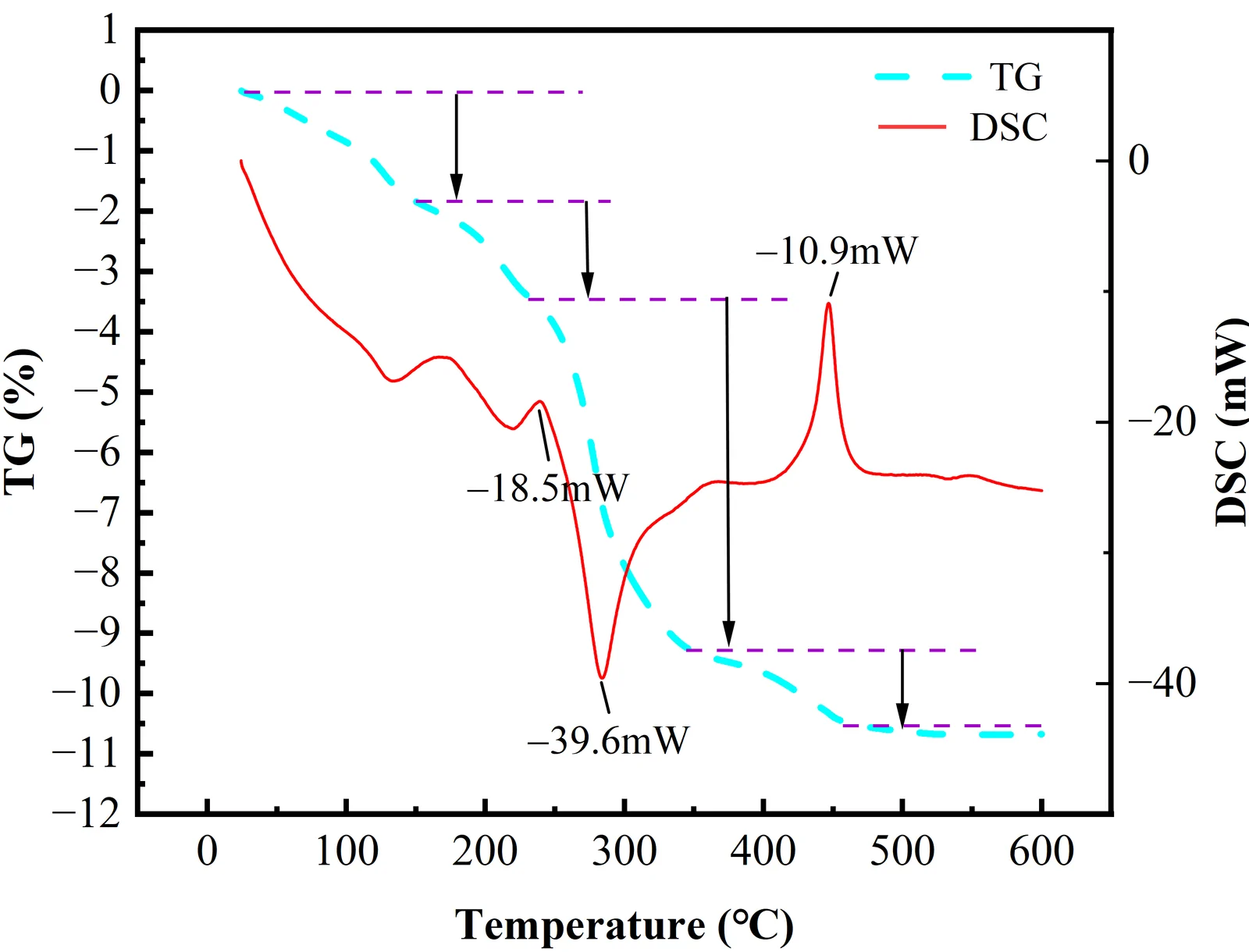
TG and DSC curves of APT during thermal decomposition under an air atmosphere.

Figure 3 presents thermogravimetric (TG) and differential scanning calorimetry (DSC) curves for APT thermal decomposition in air. The DSC curve is dominated by endothermic events throughout the 25–600 °C temperature range, with peak values ranging from 0 to −40 mW, indicating the predominantly endothermic nature of the decomposition process.

The TG curve reveals multi-stage mass loss. An initial sharp mass loss is observed, with the sample mass stabilizing only above ~500 °C. The first significant mass loss stage (25–148 °C) coincides with continuous heat absorption: this stage accounts for 2% of the total mass loss, attributed to crystallization water desorption from APT [21,23]. This multi-stage decomposition behavior is consistent with the findings of Lima et al. [28] on the thermal stability of APT derived from scheelite ore.

Between 149 and 234 °C, a second mass loss stage is observed, corresponding to a ~1.5% mass reduction. This stage likely combines continued crystallization water desorption with the initial release of ammonia from APT decomposition, consistent with previous literature reports [8].

Further heating to 344 °C reveals a third mass loss stage: the most pronounced in terms of slope and total mass loss (~4.7%). Correlating with the DSC data, this stage aligns with the largest endothermic peak (centered at 280 °C). This peak is attributed to the decomposition of APT into amorphous ammonium tungstate or hydrated tungsten oxide.

The fourth and final mass loss stage occurs from 345 °C to ~500 °C, corresponding to a further 1.3% mass reduction. After 466 °C, the sample mass stabilizes. The DSC curve exhibits a reduction in endothermic intensity near 446 °C, which corresponds to the crystallization of amorphous tungsten oxide into crystalline WO_3_. This feature indicates that the thermal decomposition of APT is essentially complete at this temperature [21].

Building on these TG and DSC findings, the behavior of APT at four characteristic temperatures—130 °C, 200 °C, 280 °C, and 400 °C—was investigated further. Complementary analyses characterized macroscopic morphology, microstructure via scanning electron microscopy (SEM), phase composition using X-ray diffraction (XRD), and particle size distribution through laser diffraction.

**Figure 4:**
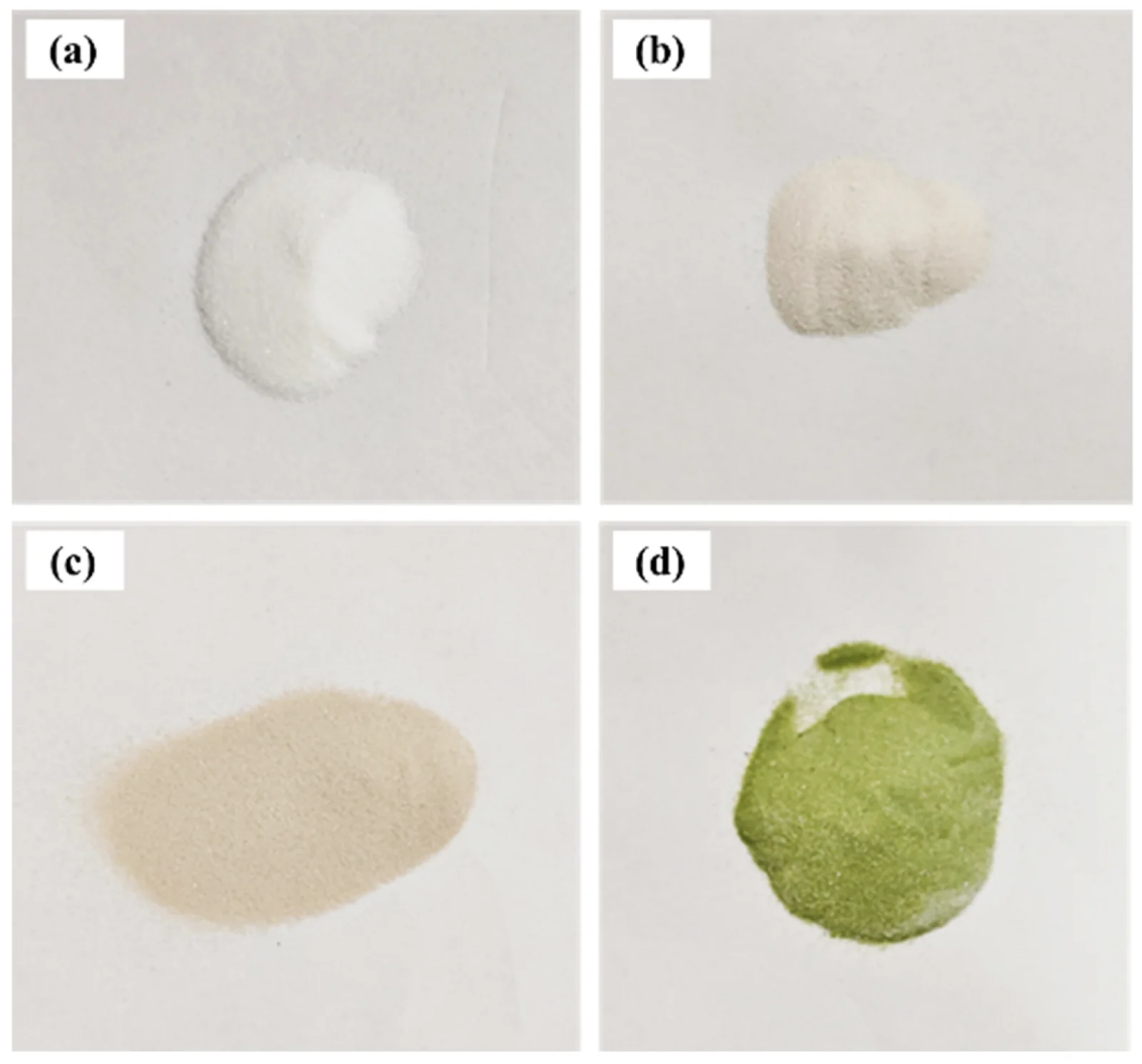
Macroscopic morphology of APT powders after heat treatment at different temperatures: (**a**) 130 °C; (**b**) 200 °C; (**c**) 280 °C; (**d**) 400 °C.

**Figure 5:**
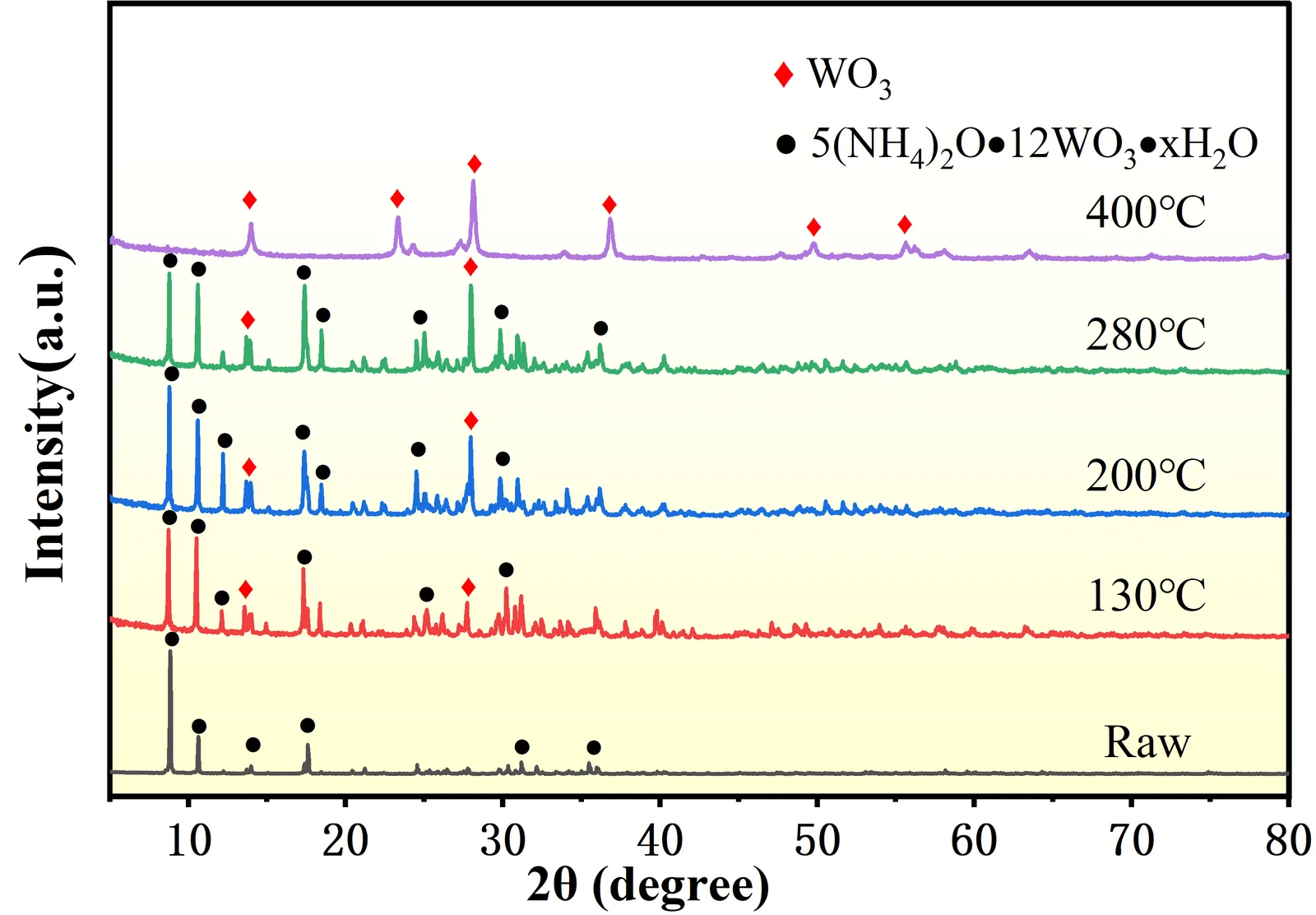
X-ray diffraction (XRD) patterns of APT powders during thermal decomposition.

Figure 4 presents the macroscopic morphology of APT powders after heat treatment at the four selected temperatures. Pronounced color changes emerge as temperature increases. As shown in the XRD patterns of Figure 5, the dehydration and deammoniation reactions progressively advance with increasing temperature. Compared with the untreated raw APT sample, the characteristic peaks of APT gradually diminish while new diffraction peaks corresponding to WO_3_ emerge and intensify at higher temperatures, confirming the partial conversion of APT to WO_3_ during thermal treatment. By 400 °C, the XRD pattern is dominated by the characteristic peaks of crystalline WO_3_, indicating that APT has undergone nearly complete decomposition into WO_3_.

The visual color changes in Figure 4a–d, from white to light gray to yellow-brown and finally green, are consistent with this progressive phase transformation from pure APT to a mixture of residual APT and decomposition products and ultimately to WO_3_.

**Figure 6:**
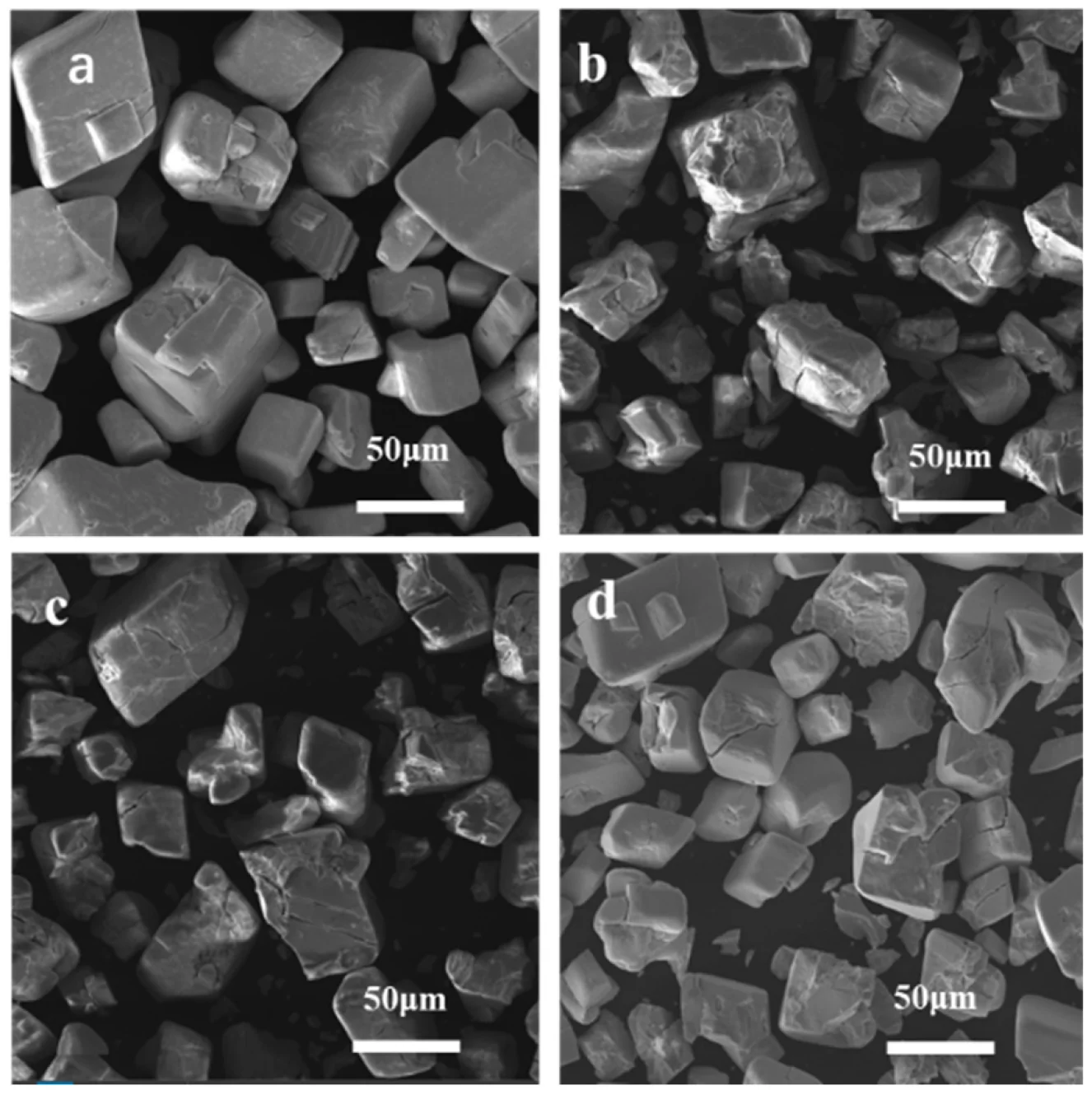
SEM morphologies of APT powders after heat treatment at (**a**) 130 °C, (**b**) 200 °C, (**c**) 280 °C, and (**d**) 400 °C.

Figure 6 presents the SEM morphologies of APT powders heat-treated at four temperatures: 130 °C, 200 °C, 280 °C, and 400 °C. Figure 6a shows uniform, granular APT crystals with smooth surfaces at 130 °C. At 200 °C (Figure 6b) and 280 °C (Figure 6c), surface defects and irregular cracks emerge, accompanied by fragmented crystals. The amorphous (NH_4_)_x_WO_y_ intermediates identified at 280 °C exhibit higher defect densities (Figure 6c)—a feature that facilitates ammonia penetration compared to crystalline WO_3_. Figure 6d at 400 °C reveals markedly improved surface roughness and more uniform morphology relative to 200 °C and 280 °C. Combined with the XRD results, these observations confirm that the powder is nearly fully converted to WO_3_ at 400 °C.

**Figure 7:**
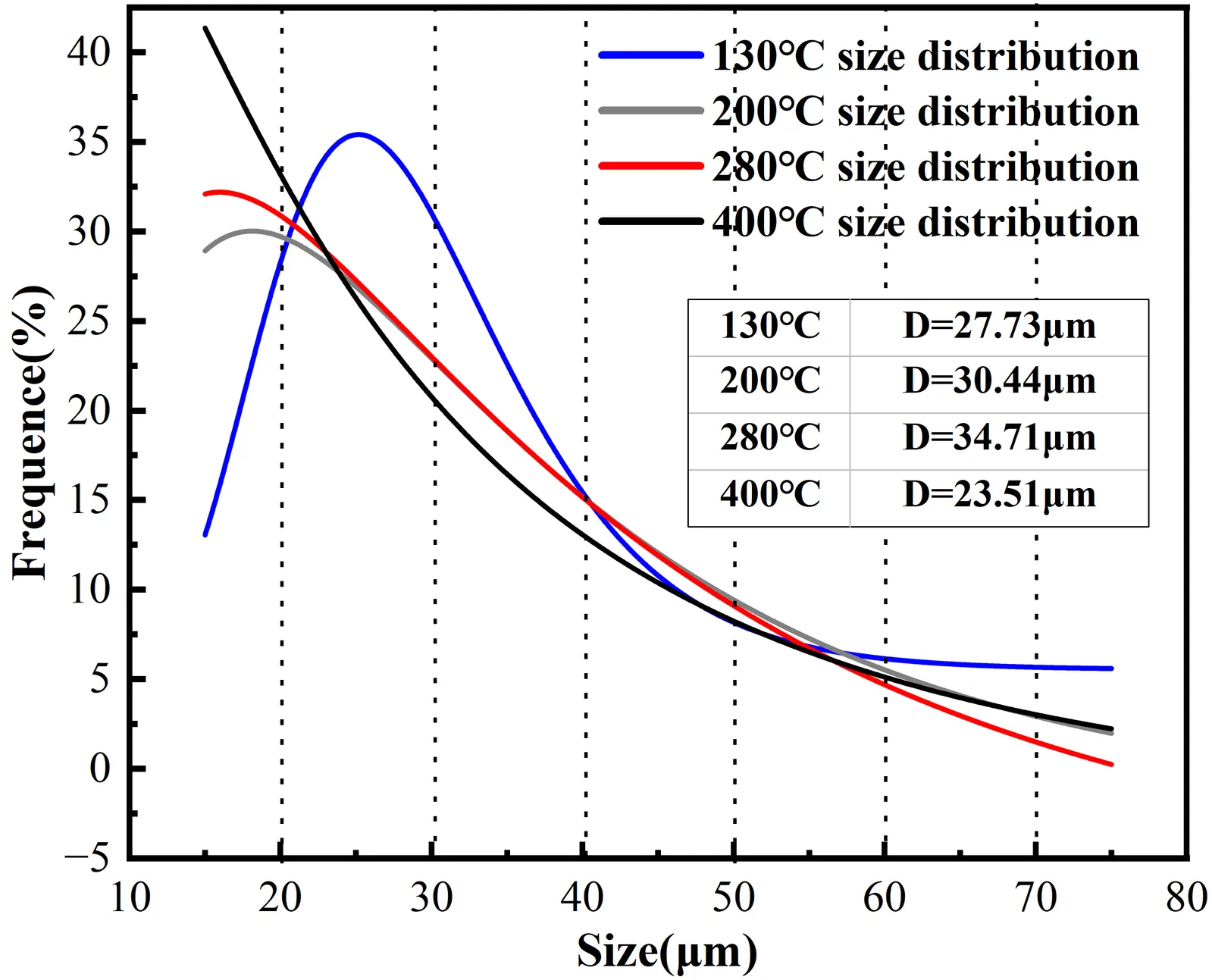
Particle size distributions of APT powders treated at different temperatures.

Figure 7 displays the particle size distributions of the APT powders at these temperatures. Despite morphological evolution, the distribution remains largely consistent across samples. Average particle sizes follow a distinct trend: 27.73 μm at 130 °C, 30.44 μm at 200 °C, peaking at 34.71 μm at 280 °C, and dropping to 23.51 μm at 400 °C.

Near 280 °C, a significant fraction of the APT crystals undergoes decomposition into amorphous ammonium tungstate or hydrated tungsten oxide intermediates. These intermediates exhibit greater reactivity toward ammonia solution than either unreacted APT or fully decomposed WO_3_. Furthermore, the powder at 280 °C exhibits surface defects and microcracks—a structural feature that enhances its specific surface area and provides additional reactive sites for ammonia infiltration and dissolution. In contrast, despite improvements in morphological uniformity at 400 °C, the resulting particles are finer, denser, and smoother, leading to reduced specific surface area and less favorable dissolution kinetics. Residual NH_4_^+^ species may also persist at 280 °C, further amplifying reactivity toward ammonia.

In summary, the decomposition process at ~280 °C optimally balances thermal decomposition extent with dissolution reactivity. This balance yields a highly soluble intermediate phase that simultaneously achieves high purity and efficient direct recovery. For this reason, 280 °C is identified as the optimal temperature for this purification step.

### 3.2. Ammonia Dissolution Process of APT

During the preparation of APT via thermal decomposition–ammonia dissolution–evaporative crystallization, impurity ions are not present exclusively in the crystallization stage. The ammonia dissolution step is equally critical, as it enables the separation of insoluble impurities through precipitation. Moreover, optimizing the dissolution temperature can significantly enhance the final direct recovery efficiency of APT [29].

APT exhibits extremely low solubility in water and dissolves relatively slowly even in ammonia solution, even under gentle heating. During ammonia dissolution, the following reactions are believed to govern the process:

(6) $$5{\left({NH}_{4}\right)}_{2}O\cdot 12{WO}_{3}\cdot 5H_{2}O\left(s\right)=10NH_{4}^{+}\left(\mathrm{aq}\right)+2{HW_{6}O_{21}}^{5-}\left(\mathrm{aq}\right)+4H_{2}O\left(l\right)$$

(7) $${HW_{6}O_{21}}^{5-}\left(\mathrm{aq}\right)+7NH_{3}\left(g\right)+3H_{2}O\left(l\right)={6WO_{4}}^{2-}\left(\mathrm{aq}\right)+7NH_{4}^{+}\left(\mathrm{aq}\right)$$

In ammonia solution, OH^−^ generated from ammonia hydrolysis consumes the HW_6_O_21_^5−^ intermediate and converts it to WO_4_^2−^. The continuous removal of this intermediate pulls the equilibrium of reaction (6) toward the product side, thereby synergistically facilitating the overall dissolution of APT in ammonia.

**Figure 8:**
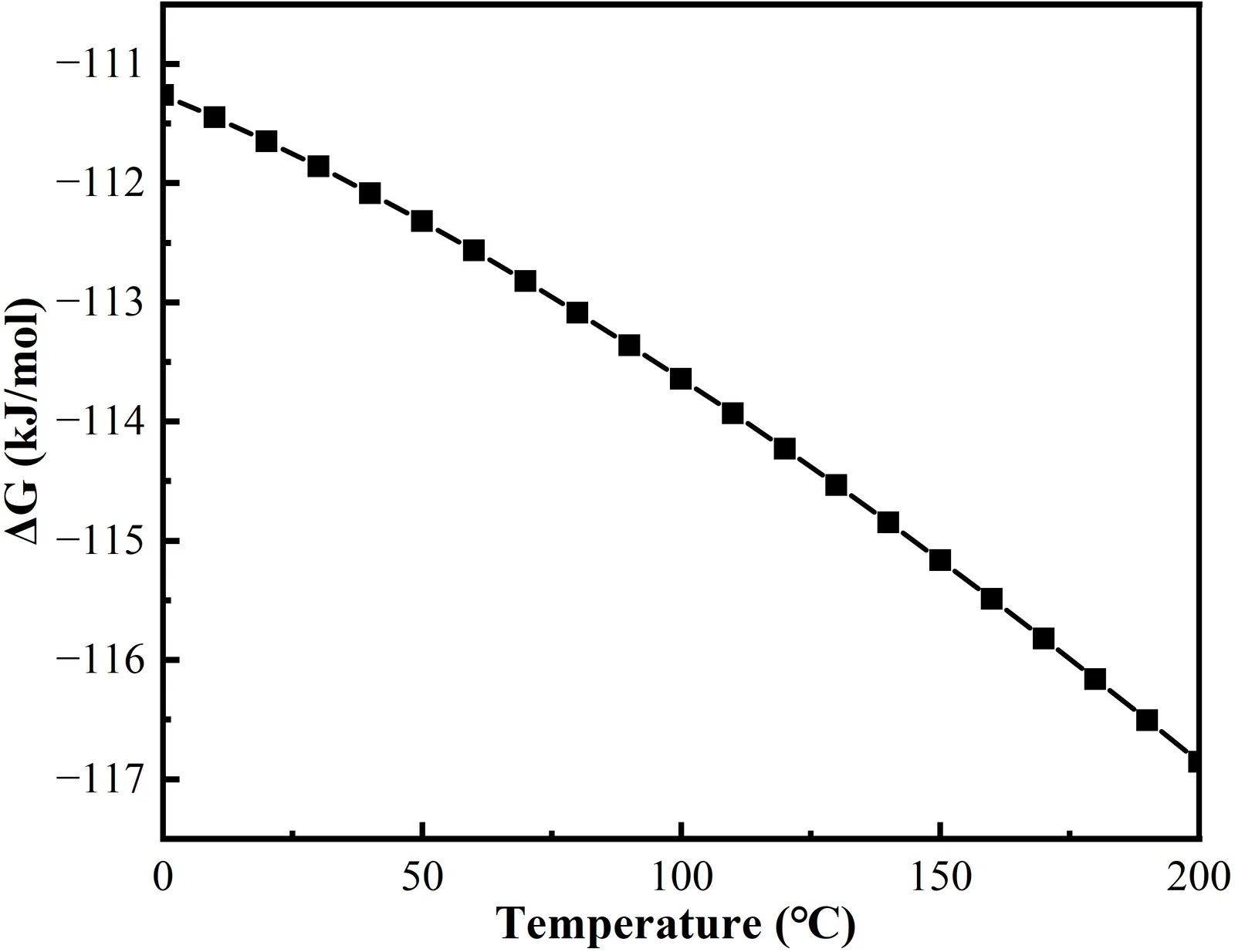
Relationship between Gibbs free energy and temperature during the ammonia dissolution of APT.

To better elucidate the dissolution mechanism, thermodynamic calculations were conducted via the built-in database of HSC Chemistry 9. Standard Gibbs free energy changes (∆G) for the key dissolution reactions were computed across a temperature range, with results presented in Figure 8.

Under standard conditions, the ∆G for the tungstate intermediate-forming reaction (Equation (6))—a critical step in APT dissolution—remains consistently negative across the studied temperature range. This confirms that this intermediate formation step, an integral part of the overall dissolution process, is thermodynamically favorable even at ambient temperature. Moreover, the increasingly negative ∆G with rising temperature reflects greater thermodynamic driving force, which correlates with accelerated reaction kinetics, thus enhancing the efficiency of ammonia dissolution.

To evaluate the effect of temperature on the ammonia dissolution efficiency of thermally decomposed APT, dissolution experiments were conducted at five temperatures: 60 °C, 70 °C, 80 °C, 90 °C, and 100 °C. After ensuring complete dissolution, the resulting ammonium tungstate solution was filtered under vacuum to isolate and quantify undissolved residues. The dissolution efficiency was calculated based on the mass loss of the undissolved solid, with results compiled in Table 1.

As summarized in Table 1, the dissolution efficiency of APT increased monotonically with rising temperature. This trend is attributed to enhanced reaction kinetics and greater thermodynamic favorability at elevated temperatures. Specifically, higher temperatures accelerate OH^−^ generation via ammonia (NH_3_) dissociation; the resulting OH^−^ readily reacts with H^+^ from water, thereby shifting the dissolution equilibrium of reaction (7) forward toward product formation and accelerating the overall process.

Thermodynamic calculations corroborate the experimental trend: as temperature increases from 60 °C to 100 °C, the standard Gibbs free energy change (∆G) for the reaction decreases from −112.57 kJ/mol to −113.60 kJ/mol, indicating greater thermodynamic favorability (i.e., more negative ∆G values). This aligns with the experimentally observed rise in dissolution efficiency.

At dissolution temperatures below ~90 °C, the process is kinetically limited: increasing temperature promotes ammonia dissociation and accelerates solid-liquid reaction kinetics. However, once temperature reaches ~90 °C, the system nears thermodynamic saturation—further temperature increases no longer enhance the thermodynamic driving force for dissolution. Instead, the rate becomes limited by interfacial mass transfer. Concurrently, ammonia volatilization intensifies, leading to a plateau in the dissolution rate. Thus, balancing dissolution performance with operational cost-effectiveness, 90 °C is identified as the optimal dissolution temperature.

### 3.3. The Segmented Evaporative Crystallization Process

According to modern crystallization theory, crystal formation proceeds through two primary kinetic stages: nucleation (the formation of microscopic crystal nuclei) and growth (the expansion of these nuclei into larger crystals) [30].

**Figure 9:**
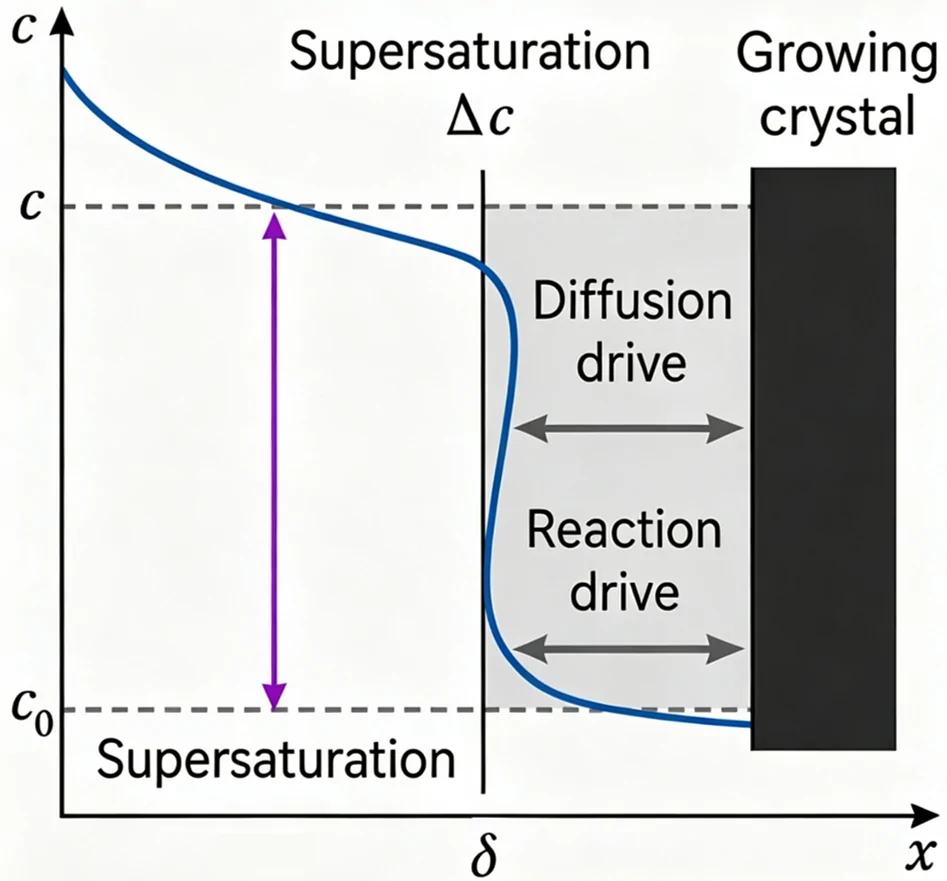
Schematic diagram of the driving force for crystal growth.

To characterize crystal growth, three key concentrations were defined as c (bulk solute concentration), c_0_ (saturation concentration), and c_1_ (concentration at the crystal growth interface). Under typical growth conditions, the concentration profile follows c > c_1_ > c_0_, as illustrated in Figure 9, which depicts the thermodynamic driving force for crystal growth.

As shown in Figure 9, a diffusion boundary layer of thickness *δ* forms around the growing crystal interface during growth. Within this layer, solute concentration decreases sharply. When *c*_1_ (interface concentration) drops to *c*_0_ (saturation concentration), dynamic equilibrium is achieved (growth rate equals dissolution rate). When *c*_1_ < *c*_0_, net crystal growth occurs as the concentration gradient drives further mass transfer.

The growth mode of crystals is determined by the relative rates of diffusion and surface reaction. When the diffusion rate (V_diffusion_) is much lower than the surface reaction rate (V_reaction_), crystal growth is diffusion-controlled. Conversely, when V_diffusion_ ≫ V_reaction_, crystal growth is surface-reaction-controlled.

Like most crystallization systems, APT crystallization proceeds through sequential nucleation and crystal growth stages, each governed by its own kinetics.

The nucleation rate *V*_1_ of APT is described by the following equation:

(8) $$V_{1}=k\;\times \;{\left(\frac{c-c_{0}}{c_{0}}\right)}^{n}=k\;\times \;{\left(\frac{\Delta c}{c_{0}}\right)}^{n}$$

Here, *c* is the bulk solution concentration, *c*_0_ is the equilibrium solubility of APT, Δ*c* is the supersaturation, *n* is the nucleation reaction order (≈3–5 for APT), and *k* is the rate constant, which depends on temperature and interfacial energy.

The crystal growth rate *V*_2_ is expressed as follows:

(9) $$V_{2}=S\times \frac{D}{\delta }\times {\Delta C}^{m}=k_{g}S{\Delta C}^{m}$$

Here, *D* is the solute diffusion coefficient, δ is the diffusion boundary layer thickness, *S* is the crystal surface area, *m* is the reaction order for crystal growth kinetics (*m* ≈ 1–2 for APT), and kg is the surface reaction rate constant.

The kinetic parameters for nucleation and growth were derived from the temporal evolution of solution concentration during evaporative crystallization. At 5 min intervals, approximately 10 mL of the supernatant was withdrawn from the crystallizer, and its mass was determined. The clear solution was then transferred into a pre-weighed container and dried to constant weight under vacuum at 80 °C. The mass of the dried solute was recorded, and the bulk APT concentration was calculated from the solute mass and the corresponding solution mass. Table 2 summarizes the time-dependent solution and solute masses, from which the bulk APT concentration *c* and supersaturation Δ*c* = *c* − *c*_0_ were calculated (*c*_0_ = 0.075 mol/L). The transition between the nucleation-dominated stage (t ≤ 35 min) and the growth-dominated stage (t > 35 min) was identified based on the derivative of supersaturation with respect to time, where *d*(Δ*c*)/*dt* dropped to ~28% of its maximum value at 35 min. Nonlinear fitting of the nucleation rate V_1_ = $k\;\times \;{\left(\Delta c/c_{0}\right)}^{n}$ and growth rate V_2_ = $k_{g}S{\Delta C}^{m}$ was performed using OriginPro 2024, where S is the crystal surface area estimated from SEM images. For the APT system, this fitting determined n = 4.2 ± 0.3 and m = 1.5 ± 0.2, with R^2^ > 0.98, confirming the higher sensitivity of nucleation to supersaturation compared to growth.

Equation (8) reveals that *V*_1_ (nucleation rate) exhibits strong dependence on supersaturation; as Δ*c* increases, *V*_1_ rises significantly. In contrast, Equation (9) shows that the crystal growth rate *V*_2_ (growth rate) is governed by both supersaturation and kinetic/diffusion factors (diffusion coefficient *D*, boundary layer thickness *δ*, and surface area *S*). Since *n* > *m* in the APT (4.2 > 1.5), supersaturation exerts a disproportionately stronger influence on nucleation than on growth. Crystallization is thermodynamically driven by supersaturation (ΔG < 0), but the dissipation of this driving force is kinetically constrained. High supersaturation enhances nucleation, while growth is limited either by solute diffusion (slow mass transfer to the crystal surface) or surface incorporation (limited active sites for attachment). Thus, the observed growth mode reflects the balance between thermodynamic driving force (ΔG < 0) and kinetic constraints.

During the nucleation stage, high supersaturation drives rapid nucleation, generating a large population of crystal nuclei. These nuclei rapidly deplete solute from the solution, limiting subsequent crystal growth and ultimately yielding fine crystals. Conversely, lower supersaturation reduces nucleation events: fewer nuclei form, allowing the solute to sustain prolonged growth and producing coarser crystals. In the growth stage, elevated supersaturation accelerates crystal growth rates, promoting enlargement; reduced supersaturation dampens growth kinetics, slowing particle expansion [16].

By independently regulating supersaturation during these two distinct stages, namely crystal nucleation and growth, the evaporative crystallization process of APT can be precisely tailored to achieve targeted particle size and high purity.

Building on the previously described kinetic distinctions between nucleation and crystal growth, the experiments were conducted to evaluate the impact of independently controlling these stages during the evaporative crystallization of APT. To isolate the influence of temperature on each stage, all other process parameters were maintained constant: linear stirring speed was fixed at 1.26 m/s, and the initial ammonium tungstate concentration was set to 70 g/L. Crystallization was performed with independent temperature regulation of the nucleation and growth stages. Pre-experiments under the same conditions (initial APT concentration 70 g/L, heating rate 2 °C/min) were carried out to find the transition point from the nucleation-dominated stage to the growth-dominated stage for the segmented evaporative crystallization. The concentration was determined by taking samples from the solution every 5 min. The saturation concentration (c_0_) of APT was measured independently as 0.075 mol/L under the conditions. The supersaturation Δ*c* = *c* − *c*_0_ was calculated, and the growth rate d(Δc)/dt was obtained. The nucleation stage was assumed to be finished when the supersaturation growth rate was less than 30% of the maximum rate. The maximum growth rate was between 30 and 35 min, and the 35 min rate was already about 28% of the maximum. Accordingly, 35 min was set as the transition time in the segmented protocol. Following crystallization, the resulting mixture was separated into solid and liquid phases, and both the APT crystals and mother liquor were subjected to detailed analysis.

**Figure 10:**
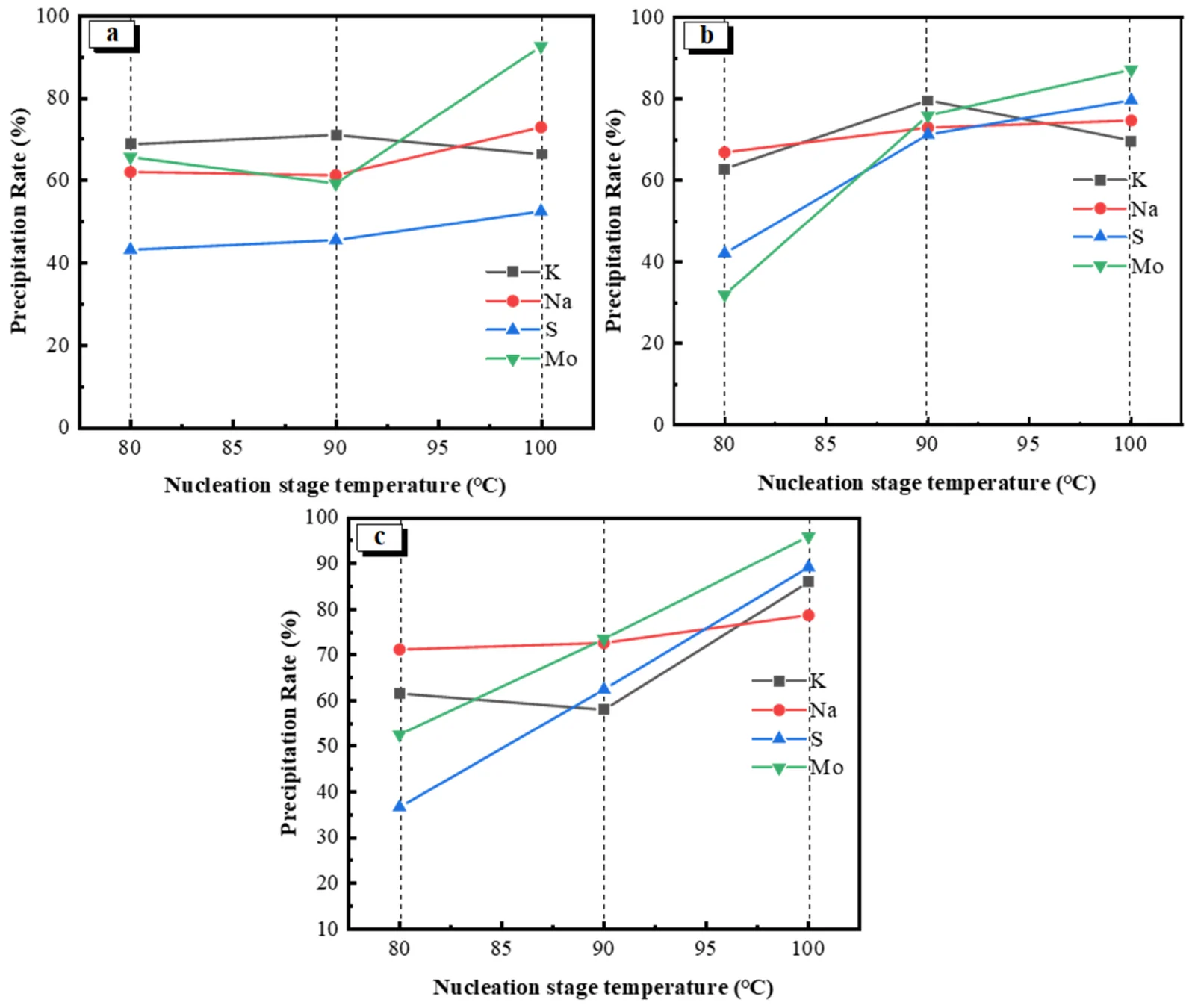
Effect of evaporation temperature on impurity exclusion during the crystal nucleation stage (stirring linear speed: 1.26 m/s; initial ammonium tungstate concentration: 70 g/L): (**a**) growth stage at 80 °C; (**b**) growth stage at 90 °C; (**c**) growth stage at 100 °C.

To investigate the effect of nucleation temperature on impurity removal, experiments were conducted where the nucleation temperature was varied while other parameters remained constant. The removal efficiencies of four typical impurity elements (Na, K, S, and Mo) in the mother liquor were quantified to evaluate this effect, with results summarized in Figure 10.

As shown in Figure 10a, the removal efficiencies of Mo, Na, and S increased monotonically with rising nucleation temperature. In contrast, the precipitation rate of K peaked at 90 °C, followed by a slight decline. Collectively, these trends indicate that a moderate increase in nucleation temperature enhances impurity removal, though this effect plateaus beyond a certain threshold. Notably, lower nucleation temperatures were found to be conducive to impurity retention in the liquid phase.

A plausible mechanism underlying this observation is as follows: lower nucleation temperatures reduce nucleation rates, limiting the number of fine crystals formed. This decreases the total surface area available for impurity adsorption, favoring impurity retention in the bulk solution. Conversely, at higher temperatures, elevated supersaturation accelerates nucleation, generating a large population of crystals with increased surface area, enhancing impurity adsorption onto the APT surface and improving precipitation rate. For potassium (K), the initial increase in removal at lower temperatures may reflect stronger adsorption affinity, while the decline above 90 °C could arise from competing processes (e.g., increased solubility of K-containing species or reduced adsorption sites due to crystal growth kinetics).

**Figure 11:**
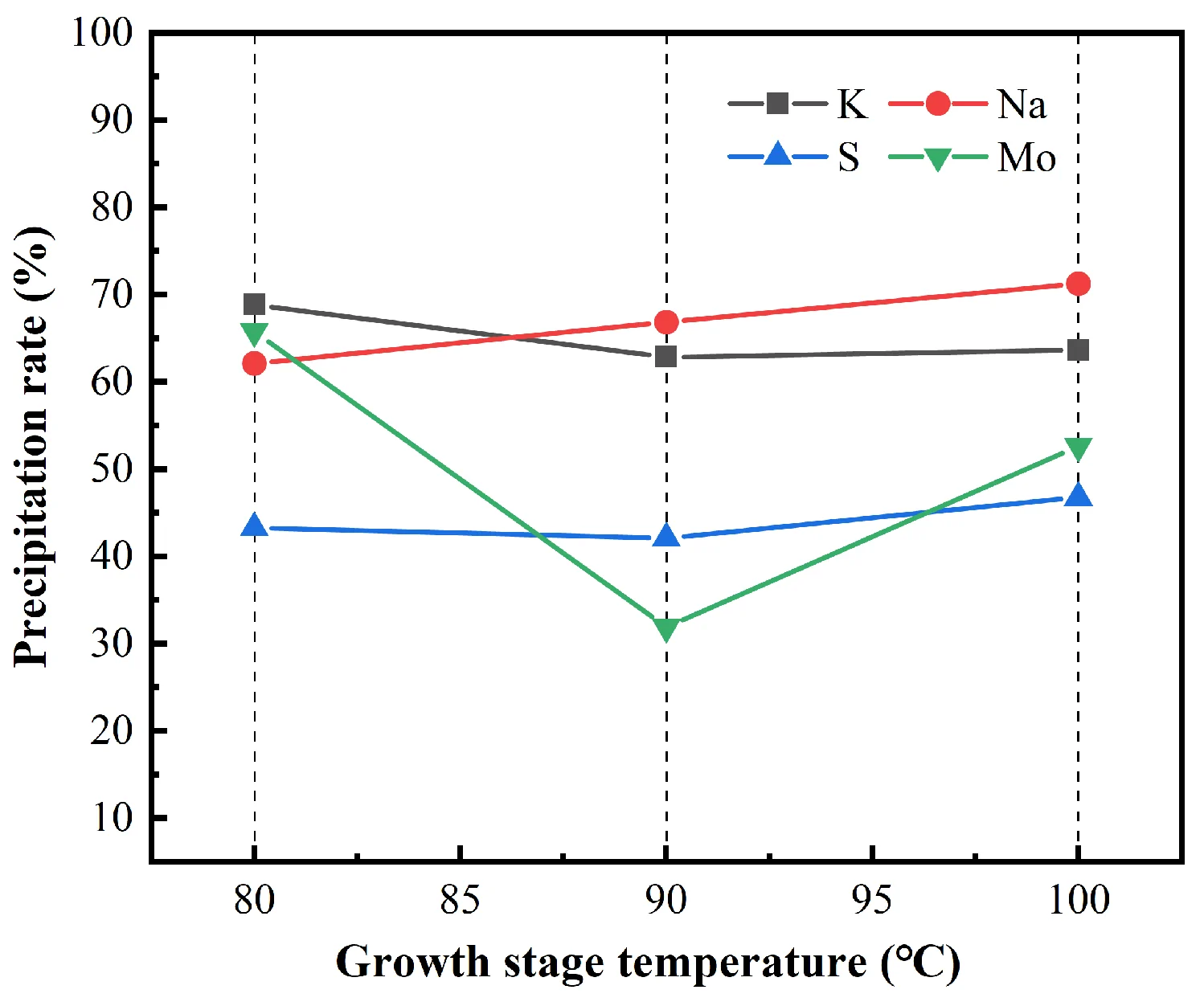
Effect of temperature during the growth stage on impurity exclusion.

Based on the previously optimal parameters, the nucleation stage was set at 80 °C, stirring speed at 1.26 m/s, and initial ammonium tungstate concentration at 70 g/L. The effect of growth temperature on impurity removal was investigated, with results summarized in Figure 11.

The overall precipitation rate of the four impurities exhibited a non-linear trend: it initially decreased, then increased with rising temperature. Specifically, Na removal rose continuously across the tested range, whereas removal rates for K, S, and Mo decreased with increasing temperature until 90 °C, after which they rebounded and began to increase again. This behavior suggests that moderate growth temperatures favor the formation of coarse, uniform crystals with fewer surface defects. Such defects act as adsorption sites for impurities; a lower defect density hinders impurity uptake onto the APT crystal surface.

To evaluate the role of stirring speed, all other parameters were fixed (nucleation temperature at 80 °C; growth temperature at 90 °C; initial concentration at 70 g/L). Stirring speeds were varied independently during the nucleation and growth stages, and the resulting APT crystals and mother liquors were subjected to comprehensive chemical analysis.

**Figure 12:**
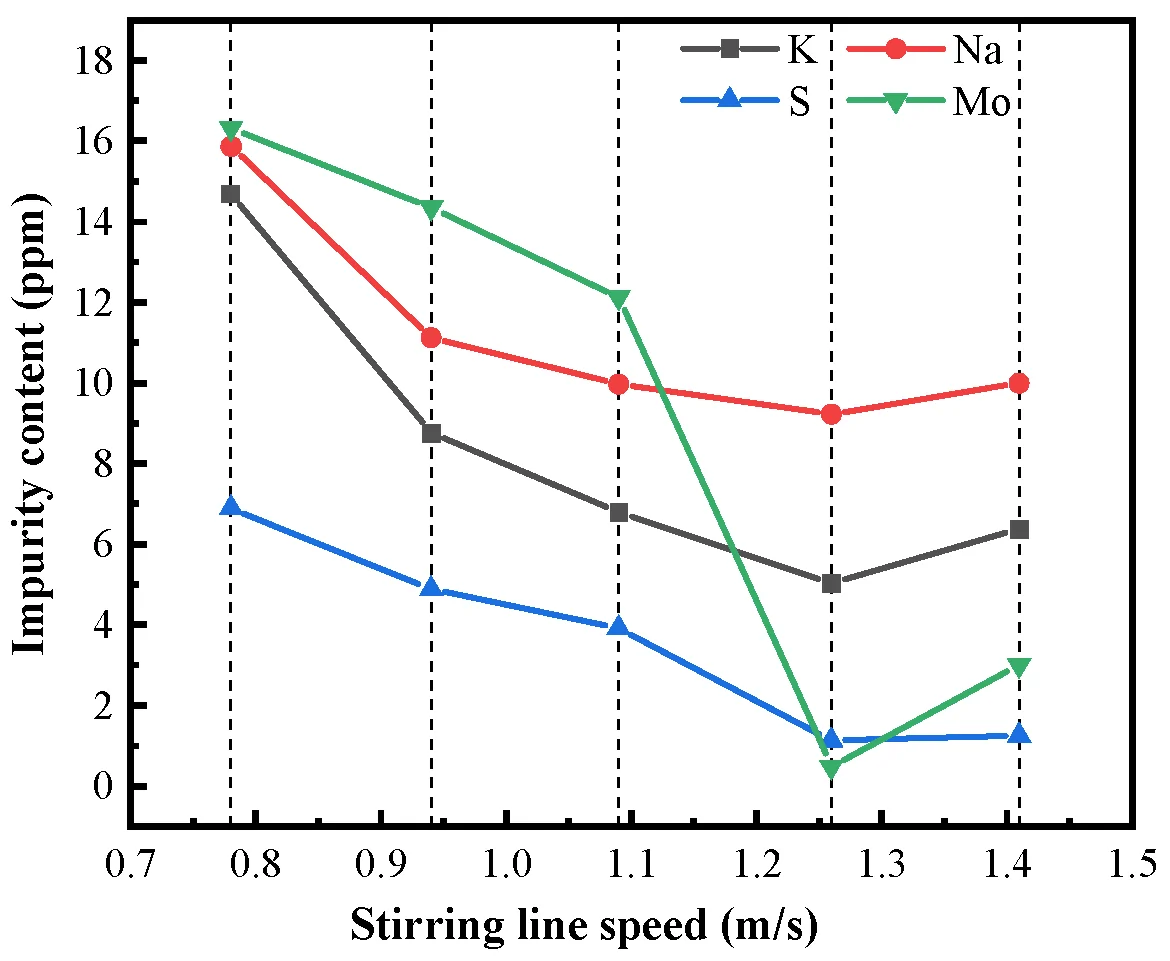
Effect of stirring speed during nucleation on impurity content in APT.

Experiments were conducted under different linear stirring speeds in the nucleation stage. The impurity content (Na, K, S, and Mo) in the final APT product was quantified, with results presented in Figure 12. The V-shaped trend observed reveals a balance between two competing impurity incorporation mechanisms: below 1.26 m/s, impurity inclusion dominates, primarily via lattice substitution (small impurity ions replacing host ions in the APT crystal structure); above 1.26 m/s, mechanical entrapment becomes the primary mechanism, driven by increased crystal fragmentation (higher stirring causes finer crystals, which are more prone to trapping impurities in their irregular surfaces).

As shown in Figure 12, impurity concentrations in the final APT product exhibited a U-shaped trend with stirring speed, reaching a minimum at 1.26 m/s. This optimal stirring rate balances two competing effects: Moderate stirring (≤1.26 m/s) enhances mass transfer by thinning the diffusion boundary layer, which homogenizes solute concentration near the crystal surface. This mitigates local supersaturation and sudden nucleation, promoting stable crystal growth. Concurrently, it reduces impurity incorporation via two pathways: minimizing lattice substitution (small impurity ions replacing host ions in the crystal structure) and decreasing surface adsorption of impurity ions (e.g., Na^+^, K^+^) by limiting crystal fragmentation. Additionally, moderate fluid shear force facilitates the desorption and diffusion of weakly adsorbed impurities from crystal surfaces back into the bulk solution, further elevating product purity. However, excessively high stirring (>1.26 m/s) induces detrimental effects. Strong shear force causes crystal fragmentation and particle collisions, generating fresh surfaces and microcracks that drastically increase specific surface area. These high-energy surfaces act as active sites for impurity re-adsorption and mechanical entrapment. Simultaneously, turbulent flow sweeps suspended impurity particles into crystals or onto newly formed surfaces, exacerbating impurity inclusions. Together, these mechanisms amplify impurity uptake at high stirring rates.

**Figure 13:**
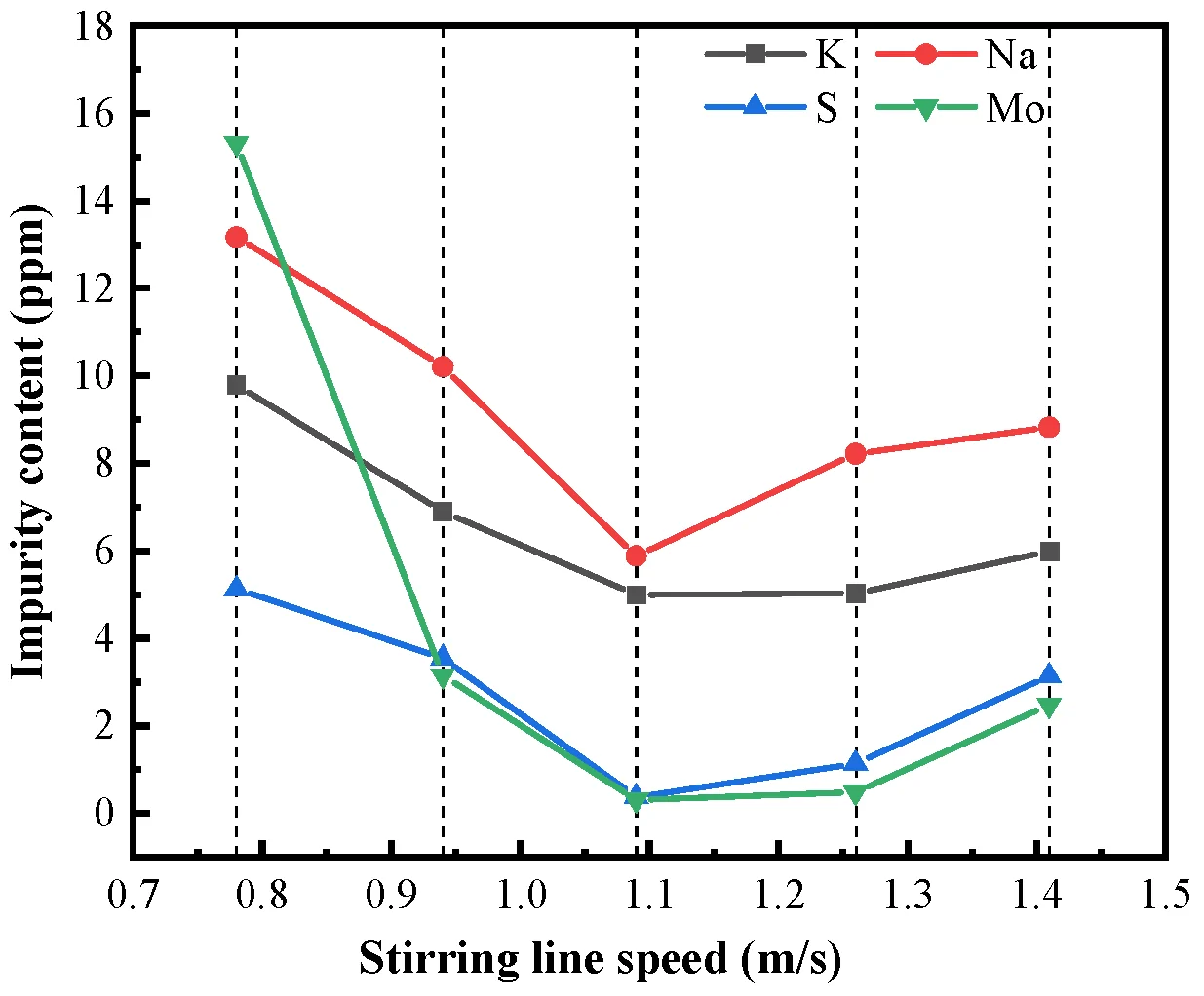
Effect of stirring speed during growth on impurity content in APT.

Guided by prior temperature optimization results, the nucleation conditions were fixed at 80 °C (temperature) and 1.26 m/s (stirring speed). Growth-stage temperature was set at 90 °C, and stirring speeds were systematically varied, and the impurity concentrations in the final APT crystals were quantified, with results summarized in Figure 13.

It can be seen that impurity levels exhibited a U-shaped trend with increasing growth-stage stirring speed, reaching a minimum at 1.09 m/s. Moderate stirring (0.78–0.94 m/s) enhances impurity removal through three synergistic mechanisms: (1) reduced Na^+^ adhesion: increased shear disrupts electrostatic interactions between Na^+^ ions and crystal surfaces, limiting lattice substitution; (2) enhanced migration of K^+^: higher fluid energy accelerates the migration of K^+^ ions from crystal defects into the bulk solution; (3) selective precipitation of co-impurities: enhanced turbulence promotes the independent nucleation of molybdenum-bearing polyoxometalates and sulfur compounds (e.g., arsenic trisulfide, As_2_S_3_), reducing their entrapment within APT crystals. Collectively, these processes suppress coprecipitation and occlusion. However, with excessive stirring (>1.09 m/s), the process induces detrimental outcomes: crystal fragmentation and collisions with impurity particles generate fresh high-energy surfaces and microcracks [31]. These sites act as active adsorption centers for impurity reincorporation, while turbulent flow sweeps suspended contaminants into crystal crevices—significantly elevating final impurity loads.

### 3.4. Crystal Morphology of APT

Based on the previously optimized parameters for segmented evaporative crystallization, the crystallization process was conducted under the precisely controlled conditions: the nucleation stage was maintained at 80 °C with a linear stirring speed of 1.26 m/s, while the crystal growth stage was regulated at 90 °C with a linear stirring speed of 1.09 m/s. The resultant APT crystals were characterized by scanning electron microscopy (SEM) for morphological analysis, laser particle size analysis (LPSA) for size distribution, and inductively coupled plasma mass spectrometry (ICP-MS) for elemental composition.

**Figure 14:**
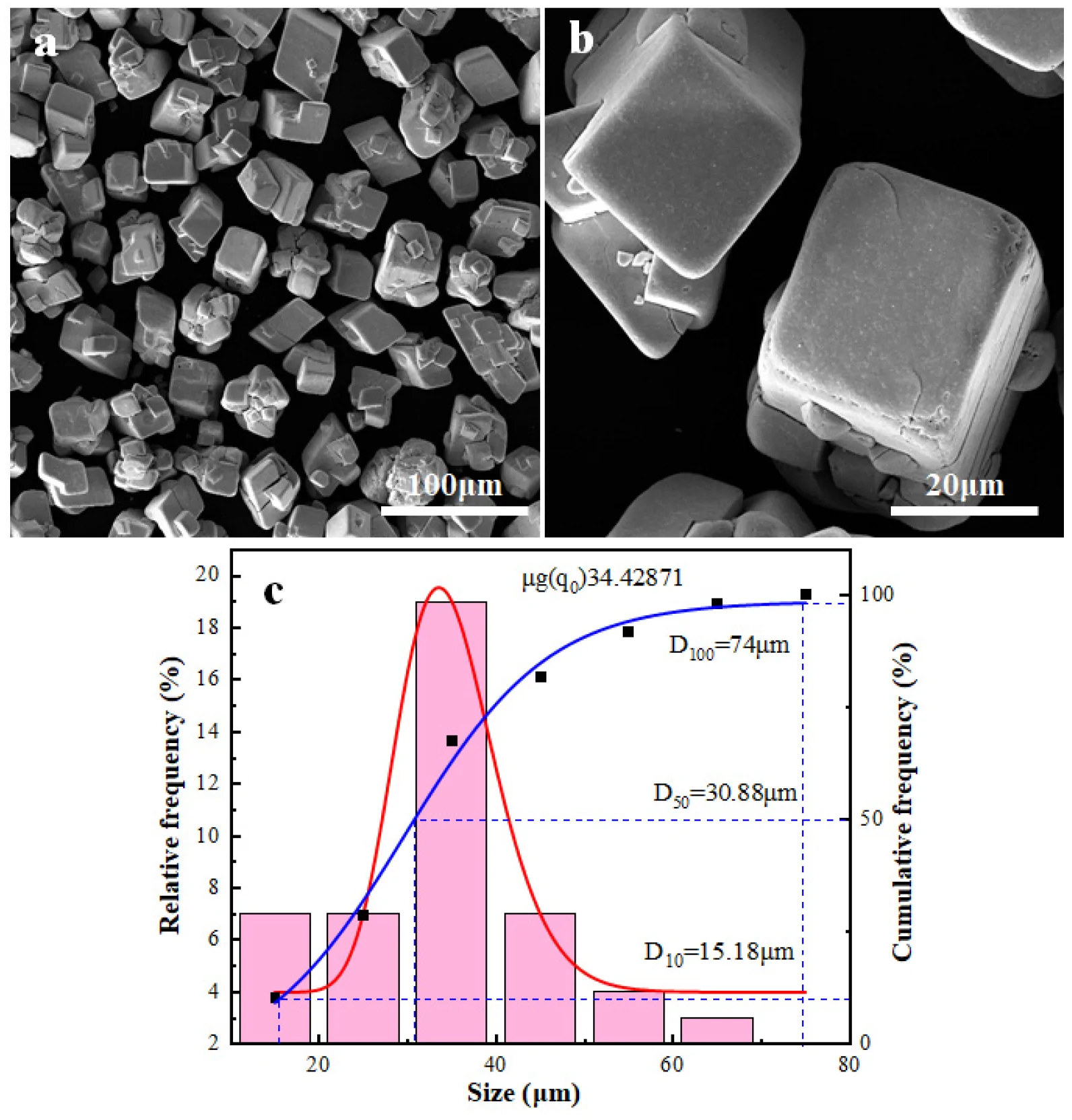
Microstructure of APT crystals obtained via segmented evaporative crystallization: (**a**,**b**) SEM images; (**c**) particle size distribution.

Figure 14a,b show the SEM images of APT crystals synthesized via segmented evaporative crystallization. The crystals exhibit smooth surfaces, minimal internal cracking, and predominantly granular or prismatic morphologies with isolated fractured fragments. This optimized morphology is attributed to the independent control of nucleation and growth conditions. During the nucleation stage (80 °C, 1.26 m/s), elevated temperature with high shear stress promotes uniform mixing, suppresses local supersaturation, and inhibits burst nucleation. During the subsequent growth stage (90 °C, 1.09 m/s), the reduced temperature lowers the nucleation driving force, while moderate stirring minimizes crystal fragmentation. These synergistic effects yield crystals with low surface energy and high structural integrity.

As shown in Figure 14c, laser particle size analysis reveals a median particle size of 30.88 μm and an average particle size of 34.43 μm. For comparison, conventional single-stage evaporative crystallization under optimized conditions yields APT crystals with an average size of only 22.6 μm and a higher proportion of fine particles [32]. The significant coarsening to 34.43 μm in the segmented process confirms that decoupling nucleation from growth effectively suppresses burst nucleation. The resulting larger crystals possess a lower specific surface area and fewer defect sites, which reduces impurity adsorption and is consistent with the 4N5 purity achieved. The detailed impurity contents are listed in Table 3. The improved purity may be attributed to the reduced adsorption sites and diminished heterogeneous nucleation. Smoother surfaces and lower crack density could reduce impurity adsorption. Furthermore, the larger particles possess a smaller specific surface area, restricting impurity seeding compared to fine-grained APT crystals.

## 4. Conclusions

(1) Thermal decomposition at 280 °C yields an intermediate with optimal ammonia solubility and a mean particle size of 34.71 μm; dissolution at 90 °C achieves an efficiency of 89.5%, balancing kinetics with thermodynamic driving force.
(2) Segmented crystallization with nucleation at 80 °C (1.26 m/s) and growth at 90 °C (1.09 m/s) produces APT with total impurities below 50 ppm, confirming that stage-specific modulation effectively suppresses impurity adsorption.
(3) The resulting APT crystals exhibit smooth, defect-minimized granular and columnar morphologies, with a median size of 30.88 μm, an average size of 34.43 μm, and a direct recovery efficiency of 73.1%, demonstrating the industrial feasibility of this single-cycle integrated process.

## Figures and Tables

**Table 1:** Dissolution rate of thermally decomposed APT at different temperatures.

Temperature (°C)	Dissolution Rate (%)
60	76.2
70	84.3
80	86.3
90	89.5
100	89.6

**Table 2:** Temporal evolution of solution concentration and solute mass during evaporative crystallization.

Time (min)	Mass of Sampled Solution (g)	Mass of Sampled Solute (g)	Concentration (mol/L)
0	10.0114	0.7778	0.0374
5	10.1607	0.8701	0.0416
10	10.2127	0.9241	0.0442
15	10.5678	1.0474	0.0488
20	10.497	1.1008	0.0520
25	10.5033	1.32945	0.0643
30	10.8347	1.6478	0.0796
35	11.0796	1.8472	0.0888
40	11.016	1.8802	0.0914
45	9.9775	1.8941	0.1040

**Table 3:** Impurity contents in APT crystals obtained by segmented evaporative crystallization.

Impurity	Content (ppm)	Impurity	Content (ppm)
K	~5.2	Bi	~0.1
Na	~6.0	Ca	~8.8
S	~0.1	Co	~0.1
Mo	~0.3	As	~3.9
Al	~4.8	Cr	~2.1
Mn	~0.6	P	~4.7
Mg	~2.1	Pb	~0.2
Cu	~0.1	Cd	~0.1
Fe	~6.0	Ni	~2.0
V	~1.0	Si	~0.2
Ti	~1.0	Sb	~0.2

## Data Availability

The original contributions presented in this study are included in the article. Further inquiries can be directed to the corresponding authors.
